# Book Reviews

**Published:** 1905-08

**Authors:** 


### BOOK REVIEWS.

---

PHARMACOLOGY OF THE FLUID OF EXTRACTS. By John S. Wright, Prepared Especially for Students of Medicine. Published from the Research Department of Eli Lilly & Co. Indianapolis, Ind. 1905.

This little work contains a wealth of information concisely stated concerning a very important class of drug preparations. The trivial and botanical names of the substances from which the extract is derived, dose, constituents and physiological action are given. In addition there is an appendix containing sections on the general principles of incompatibility of drugs, poisons and antidotes, metric system of weights and measures, table of equivalents, table to assist the beginner in prescribing liquids, rule for dosage, rule for comparing the Centigrade and Fahrenheit scales, Latin phrases and abbreviations used in prescriptions, Latin genitive case endings, symbols and signs used in prescriptions, temperature of the body, relation of pulse and temperature, average frequency of pulse at different ages in health, respiration at various ages. The price of the book, leather bound, is fifty cents.

---

### UNCINARIASIS IN PORTO RICO.

In their report of December, 1904, upon the above disease, the Commission records a number of cases cured, the hookworm being destroyed and removed by the purgative and thymol treatment. The hemoglobin percentage was raised to normal, or practically so, by the use of Pepto-Mangan (Gude), which preparation was uniformly employed for the anemia. The report is very interesting and the results striking.

---

A new law forbids the publication in Norwegian newspapers of advertisements to further the sale of any and all foreign patent medicines.

